# Neoadjuvant intraperitoneal chemotherapy followed by radical surgery and HIPEC in patients with very advanced gastric cancer and peritoneal metastases: report of an initial experience in a western single center

**DOI:** 10.1186/s12957-018-1363-0

**Published:** 2018-03-22

**Authors:** Bianca Escorel Costa Fava, Wilson Luiz da Costa, Maria Luiza L. Medeiros, Marina Sonagli, Héber Salvador de Castro Ribeiro, Alessandro L. Diniz, André L. Godoy, Igor C. de Farias, Victor Hugo Fonseca de Jesus, Maria Dirlei F. S. Begnami, Felipe J. F. Coimbra

**Affiliations:** 1 Department of Abdominal Surgery, A. C. Camargo Cancer Center, Rua Antonio Prudente, 211, Liberdade, Sao Paulo, CEP 01501-900 Brazil; 2 Departament of Medical Oncology, A. C. Camargo Cancer Center, Sao Paulo, Brazil; 3 Department of Pathology, A. C. Camargo Cancer Center, São Paulo, Brazil

## Abstract

**Background:**

The association of preoperative systemic and intraperitoneal chemotherapy has been described in Eastern patients with very good outcomes in treatment responders. The aim of this paper is to describe the initial results of this multidisciplinary regimen in gastric cancer patients with very advanced peritoneal metastases.

**Case presentation:**

We present here the first four cases who received the treatment protocol. They had a baseline PCI between 19 and 33. Two patients had received systemic chemotherapy prior to this regimen. Three of them had significant response and were taken to cytoreductive surgery, while one patient who had 12 cycles of chemotherapy previously showed signs of disease progression and subsequently died. There was no significant postoperative morbidity, and three patients remain alive, two of them with no signs of recurrence.

**Conclusion:**

Systemic and intraperitoneal chemotherapy led to a marked response in peritoneal disease extent in our initial experience and allowed three of four patients with very advanced disease to be treated with cytoreductive surgery.

## Background

Patients with advanced gastric cancer present with peritoneal metastases in about 30% of cases [1] and up to 50% of those treated with curative intent will develop relapse in the peritoneum. Standard treatment for these individuals is systemic chemotherapy, but median survival in this scenario is poor, around 3 to 6 months in most studies [2], reaching a little over 1 year in a recent Eastern trial [3].

Cytoreductive surgery (CRS) followed by hyperthermic intraperitoneal chemotherapy (HIPEC) for gastric cancer patients with peritoneal metastases has been associated with improved survival in a selected group of patients both in an Eastern [2] and in a large French multicenter series [4]. The results from both studies have emphasized the importance of patient selection, as the ones with the best results were treated with preoperative systemic chemotherapy, had limited peritoneal dissemination, measured by a low peritoneal cancer index (PCI), and were treated with a complete cytoreduction [4].

The association of neoadjuvant intraperitoneal and systemic chemotherapy has been investigated recently and seems to be a very important tool for patient selection. In a large Eastern series, individuals who had negative cytology after this preoperative regimen and were treated with CRS + HIPEC had improved survival compared to those with positive cytology [5]. The addition of laparoscopic HIPEC (L-HIPEC) and more effective systemic chemotherapy to this multidisciplinary treatment, labeled as bidirectional intraperitoneal and systemic chemotherapy (BISIC), has led to more significant response rates and improved survival in this set of patients [1].

The aim of this study was to report the first four consecutive cases of gastric cancer patients who presented with advanced disease and disseminated peritoneal metastases and were treated with L-HIPEC and BISIC, followed by CRS + HIPEC.

## Methods

This is a retrospective, single-center case series based on routinely collected data extracted from patients’ electronic charts. This paper was written in accordance with CARE guideline for case reports [6].

The inclusion criteria were diagnosis of synchronous metastatic gastric cancer with peritoneal dissemination as the sole site of metastatic disease and treatment with BISIC (as described below) between October 2015 and August 2017 (Table 1).

**Table 1:** Clinical characteristics of the four patients treated for gastric adenocarcinoma metastatic to the peritoneum with BISIC

Age	Sex	Histology	Baseline PCI	PCI at re-staging	PCI at CRS	Survival	Status
29	M	Mixed-type adenocarcinoma	20	15	17 (no CRS performed)	16	DEAD
34	F	Signet-ring cell adenocarcinoma	33	25	2	27+	AWOD
55	F	Signet-ring cell adenocarcinoma	25	17	12	15+	AWD
57	M	Signet-ring cell adenocarcinoma	Not available	19	13	13+	AWOD

*AWOD* alive without disease, *AWD* alive with disease

Treatment was adapted according to Yonemura’s original protocol (2006), which he later modified, adding more effective systemic chemotherapy and with a different dosage of the intraperitoneal drugs [1, 7]. As S-1 is not currently available in Brazil, Capecitabine was used instead of S-1. Briefly, during the first laparoscopy, before any treatment, extensive intraperitoneal lavage (EIPL) [8] and L-HIPEC (Cisplatin 30 mg/m2 + Docetaxel 30 mg/m2, for 1 h at 42 ^o^C) were performed. At the end of the procedure, a peritoneal port-a-cath (DistricAth®, Districlass Médical, France) was placed with its tip directed toward the cul-de-sac. After a 15-day period of rest, patients initiated normothermic chemotherapy. On day 1, Cisplatin 30 mg/m2 and Docetaxel 30 mg/m2 were infused into the peritoneal cavity for 2 h after adequate pre-medication. On day 8, Cisplatin 30 mg/m2 and Docetaxel 30 mg/m2 were given intravenously in separate bags according to standard infusion protocols. Capecitabine 850 mg/m2 PO twice a day was administered from day 1 to day 14. Cycles were repeated every 21 days.

Treatment strategy consisted on repeating BISIC cycles three times, followed by CT scans, endoscopy, and a new laparoscopy. According to the surgical findings, another three cycles of BISIC was performed, or patient was taken to CRS, which included gastrectomy + D2-lymphadenectomy, resection of peritoneal lesions, and HIPEC.

During treatment, patients were followed for toxicity at least once per cycle, and response evaluation with endoscopy and CT scans were performed every three cycles. After cytoreductive surgery, CT scans were repeated every 2–3 months.

All patients signed informed consent after extensive discussion with patients and relatives regarding potential benefits and risks of the treatment. Toxicity was graded according to Common Toxicity Criteria (CTC) 4.0.

## Case presentation

### Case 1

A 34-year-old female patient was admitted in September 2015 at our service. She complained of epigastric pain and reported a 13-kg weight loss. Her weight at this time was 39 kg. An upper endoscopy revealed a large gastric tumor that extended from the posterior wall of the greater curvature in the fundus to the gastric antrum. Biopsy confirmed the diagnosis of a poorly differentiated adenocarcinoma. Thoracic and abdominal CT scans were then performed and showed gastric wall thickening in the fundus, mild ascites, enlarged perigastric lymph nodes, and peritoneal nodules. CEA and CA 19-9 levels were 6.4 ng/mL and 555.4 U/mL, respectively.

A staging laparoscopy demonstrated multiple peritoneal nodules in the right and left diaphragm, greater and lesser omentum, pelvis, and parieto-colic gutters. PCI count was 33. Ascites was considered to be moderate, and its cytology was positive for the presence of free cancer cells. Biopsy of two nodules confirmed the diagnosis of adenocarcinoma. After informed consent was obtained, the patient started the protocol described in the “Methods” section.

Re-staging CT scans demonstrated a decrease in gastric wall thickening, ascites, and in the number of peritoneal nodules. A new laparoscopy showed a decrease in the number of peritoneal nodules, but the PCI count remained high (20). Cytology of the peritoneal wash, however, was negative, as were the biopsy of one nodule in the right diaphragmatic peritoneum. After multidisciplinary discussion, we opted to treat the patients with three more BISIC cycles.

**Fig. 1:**
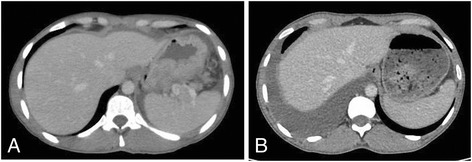
Abdominal CT showing gastric wall before (**a**) and after (**b**) the BISIC regimen. A significant response with regression of gastric wall thickening can be observed

After six cycles, the patient had regained her weight (50 kg), and her CT scans showed a significant reduction both in gastric wall thickening and peritoneal nodules (Fig. 1). Her CA 19-9 was 13.1 U/mL, and her CEA level was below detection level. During treatment with BISIC, patient developed mild toxicities, including G1 nausea, vomiting, fatigue, alopecia, decreased appetite and diarrhea, and G2 constipation and infection (upper respiratory tract infection). Among hematological toxicities, only grade 2 anemia was observed. There were no dose reductions or treatment delays due to toxicity. No serious adverse event was reported.

**Fig. 2:**
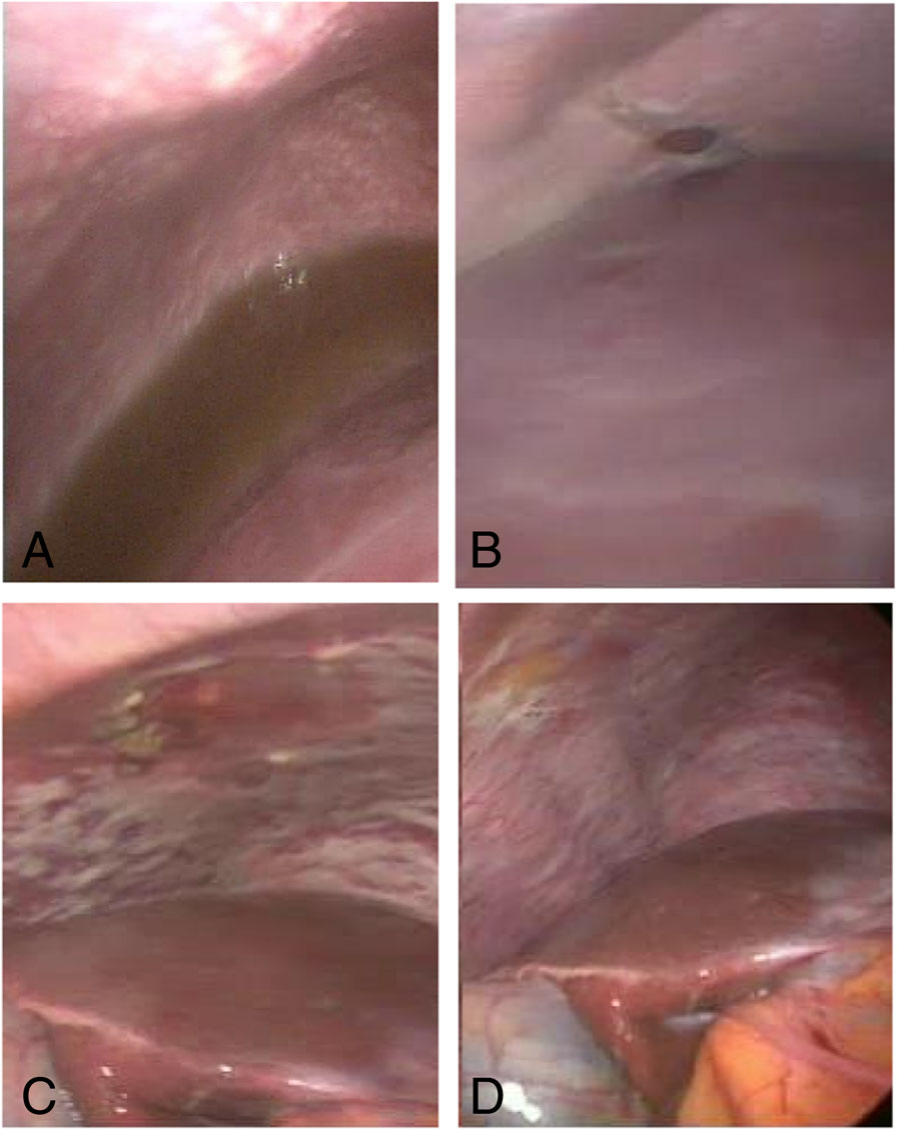
Peritoneal surface before and after systemic and intraperitoneal chemotherapy. **a** Case 1, before treatment; **b** case 1, after L-HIPEC and six cycles of BISIC regimen; **c** case 2, before treatment; **d** case 2, after L-HIPEC and six cycles of BISIC

The patient was then taken to surgery in June 2016. A new staging laparoscopy identified no peritoneal lesions (Fig. 2). We proceeded to a laparotomy, and a total gastrectomy with D2-lymphadenectomy was performed. Peritoneal areas in the right and left diaphragm, in the pelvis, and in the small bowel mesenterium were resected. EIPL was performed after resection, followed by HIPEC with Docetaxel 30 mg/m^2^ and Cisplatin 30 mg/m^2^ for 1 h. She had an uneventful recovery and was discharged on the tenth postoperative day.

The peritoneal wash cytology was negative and the pathology report showed only acellular mucin and rare epithelial cells with nuclear atypia in the gastric body and antrum’s mucosa and submucosa. The tumor bed measure was 10 × 4.5 cm. No lymphatic, perineural, or vascular invasion was identified. All margins were negative, and there were no metastases in the 38 dissected lymph nodes. Peritoneal wash cytology was negative, and all peritoneal areas that were resected had no signs of viable disease. Pathological staging was ypT1b ypN0 ypM0 (pathologic TNM I). Response to chemotherapy in the examined tissue was characterized as 5% of viable tumor cells and 95% of fibrosis.

After surgery patient was submitted to five additional cycles of capecitabine 750 mg/m2 PO twice a day from day 1 to day 14 every 21 days. At last follow-up (January 2018), she was asymptomatic and exams showed no evidence of disease.

### Case 2

A 55-year-old female presented in September 2016 with a long history of dyspeptic symptoms and an upper endoscopy that showed a Borrmann type IV lesion in the gastric body with a biopsy of signet-ring cell adenocarcinoma.

Staging was performed first with thoracic and abdominal CT scans, which showed diffuse gastric wall thickening and signs of peritoneal metastases. A staging laparoscopy revealed multiple peritoneal metastases, with a PCI count of 25.

**Fig. 3:**
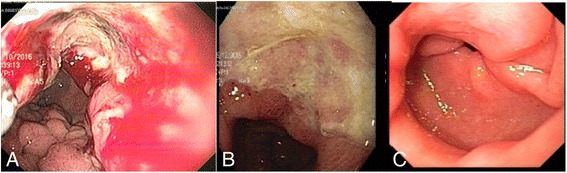
Endoscopic aspect of gastric lesion in case 2 **a** at diagnosis, **b** after three cycles of BISIC, and **c** after six cycles of BISIC demonstrating significant response to treatment

After three cycles of treatment, re-staging endoscopy demonstrated a significant response to treatment, as no ulcerated lesions remained, only a fibrotic and substenotic area in the body-antrum transition (Fig. 3). Furthermore, CT showed a regression in the thickening area. A new laparoscopy was performed in February 2017, which revealed the presence of remaining peritoneal metastases, and a total PCI of 17. The recommendation as in the previous case was to maintain the BISIC regimen and re-evaluate after three more cycles.

After the sixth cycle, re-staging with endoscopy and CT identified the same signs of response to chemotherapy. During treatment, the patient presented somewhat more toxicity in comparison to the previous patient. G1 vomiting, peripheral neuropathy, alopecia, and decreased appetite were noticed. Moreover, G2 nauseas, fatigue, abdominal pain (due to chemical peritonitis), infection (upper respiratory tract infection), and myalgia were verified. Dose reductions of the intraperitoneal component of BISIC (due to chemical peritonitis) and dose delays were deemed necessary. Severe toxicities were not observed.

A new laparoscopy was performed in June 2017 and showed less spread of peritoneal nodules, with a PCI count of 12. Cytoreductive surgery was then performed, with a total gastrectomy, D2-lymphadenectomy, resection of the diaphragmatic peritoneum, and nodules in the small bowel mesentery. Three of these nodules were sent to frozen section biopsy, with no signs of viable disease in any of them. HIPEC followed, with Cisplatin 30 mg/m^2^ and Docetaxel 30 mg/m^2^, perfused for 1 h.

This patient also had an uneventful recovery and was discharged from the hospital on the eleventh postoperative day. The pathology report described a signet-ring cell adenocarcinoma in the stomach distal body, with serosa infiltration and 4 metastatic lymph nodes, a total of 23 dissected. Regarding the peritoneal nodules, viable disease was detected in the round ligament and in one small bowel implant. (ypT3 ypN2 ypM1—pathologic TNM IV). She received four cycles of adjuvant chemotherapy (FOLFOX), from a planned total of six cycles. She developed peritoneal recurrence 3 months later and is now under treatment with a second-line chemotherapy regimen.

### Case 3

The next two cases encompass subjects with a different treatment background. The first was a 29-year-old male, admitted in December 2015, with a history of epigastric pain and a 6-kg weight loss. He had an upper endoscopy that showed a Bormann IV lesion in the gastric body and a biopsy of mixed-type adenocarcinoma. Diffuse peritoneal metastases were identified on abdominal CT and on a PET-CT.

A staging laparoscopy confirmed multiple peritoneal metastases, with a PCI count of 20. The patient received systemic chemotherapy, with 12 cycles of modified DCF (Docetaxel, Cysplatin, and 5-Flouracil). After multidisciplinary discussion, a new laparoscopy was performed and PCI was 15. L-HIPEC was then administered with Cisplatin 30 mg/m^2^ and Docetaxel 30 mg/m^2^, followed by three cycles of BISIC. The following staging procedure showed a PCI count of 17. Due to this finding of disease progression, surgery was not performed and the patient was started on second line of chemotherapy, with Paclitaxel and Ramucirumab. He had disease progression in the second cycle and died due to complications related to the tumor in April 2017.

### Case 4

Case 4 refers to a 55-year-old male, who had a different treatment background as well. He was admitted at our service in October 2016 with a 6-month history of epigastric pain and a 15-kg weight loss. His upper endoscopy revealed an infiltrative lesion in the upper body of the stomach, which resembled *linitis plastica*. Biopsy revealed a signet-ring cell adenocarcinoma. He had a previous abdominal CT with findings of diffuse peritoneal metastases and had been treated with eight cycles of FLOT (5-Fluoracil, Leucovorin, Oxaliplatin, and Docetaxel) at a cancer service in his hometown.

He underwent a staging laparoscopy in December 2016, which confirmed the peritoneal metastases with a PCI count of 19. L-HIPEC was performed as described in the previous two cases and the intraperitoneal port-a-cath positioned. After two cycles of BISIC, also administered as previously described, a new laparoscopy was performed in April 2017 and a PCI count of 13 was identified. A midline incision followed and a total gastrectomy with D2-lymphadenectomy was performed, along with the resection of the subdiaphragmatic and pelvic peritoneum, in which there were some fibrotic areas that could have residual disease. HIPEC was also administered as in the other two patients. Pathology showed a residual mixed-type adenocarcinoma invading the gastric submucosa, 17 lymph node metastases out of 22 dissected nodes and 30% of viable tumor cells in the stomach. All peritoneal areas showed no residual disease. (pT1b pN3b pM0––pathologic TNM IIB). His recovery had no significant events, and he was discharged on the twelfth postoperative day. He underwent six cycles of chemotherapy (FOLFOX) in his home town and now is on follow-up, with no signs of recurrence.

## Discussion

We report in this series the first four cases of gastric cancer patients with very advanced peritoneal disease who were treated with a multimodality regimen that included systemic and intraperitoneal chemotherapy in a recently nominated regimen known as BISIC [1], followed by cytoreductive surgery and HIPEC, in a single Western cancer center. The most important findings in these cases were the lack of postoperative morbidity and the significant response in peritoneal dissemination associated with treatment, which turned patients who would be candidates for palliative chemotherapy only into candidates for a more radical therapy and a chance for improved survival.

Three of the four patients reached a significant response that allowed them to be treated with a complete cytoreductive surgery and also received HIPEC with Docetaxel and Cisplatin for 60 min. The association of CRS and HIPEC has been proven superior to CRS alone in a Chinese randomized trial with 68 patients, in which the multimodality group had a median survival of 11 months compared to 6.5 months in the surgery only group. These poor numbers in both groups may be related to patient selection, as 42% of individuals in this study did not have a complete CRS with no residual macroscopic disease (CC-0) [9]. This trial confirmed the findings of a large French multicenter study that subjects who are candidates for this multimodality regimen should receive preoperative chemotherapy, should have low disease burden, expressed in PCI count, and should receive a CC-0 CRS as a mandatory step of their treatment [4].

Canbay et al. first described the use of neoadjuvant intraperitoneal chemotherapy combined with systemic chemotherapy in a large single-center series in Japan, with 194 patients. Out of these individuals, 152 (78%) were classified as responders, a classification that at the time included patients whose cytology became negative after the administration of two cycles of intraperitoneal Cisplatin and Docetaxel and six cycles of S-1. These responders were then taken to CRS and HIPEC, and the median survival of those who received CC-0 surgery was 18 months [5], which was an improvement compared to the results of chemotherapy alone, even in Eastern studies [10].

This regimen was later modified by the same group, and the concept of a laparoscopic hyperthermic intraperitoneal chemotherapy perfusion (L-HIPEC) was introduced. Its main advantage would be a minimally invasive procedure with direct vision, which would allow for a more valid assessment of the peritoneal disease extent in the peritoneal cavity. Also, a more effective systemic chemotherapy regimen was adopted, with the use of Cisplatin and Docetaxel intravenously associated with the intraperitoneal regimen (BISIC). This treatment has been described in detail in the literature [1, 11]. The most important benefit of this association seems to be a higher response rate in peritoneal disease extent. In a study of this regimen in 105 patients, the association of L-HIPEC and BISIC led to a significant change in PCI, as 44% of patients had baseline PCI under 12, compared to 67% after treatment. Also, 66% of all subjects experienced a decrease or complete disappearance in peritoneal metastases in the re-staging laparoscopy [12]. We identified this response in three of our patients, with decreases of 100, 52, and 31%, with numbers that were obtained in open surgery and that could have been undervalued in the staging laparoscopies. The less significant response and the disease progression findings were identified in the two patients who had systemic chemotherapy prior to L-HIPEC and BISIC. Although sample size was too small to draw any conclusions, it is possible that the performance of this prior treatment could have helped induce drug resistance.

Another aspect that should be highlighted is the very low morbidity associated with the procedure. In the study above, no patient developed grade IV and V toxicity and only four had grade III events after L-HIPEC and BISIC. Our cases had similar results, with no grade III, IV, or V events after chemotherapy and no postoperative complication. We certainly do not expect zero morbidity in future cases, but that resembles our previous results with adjuvant HIPEC, in which no patient developed organ insufficiency and there was no mortality [13]. Very similar results have been recently reported in an American cancer center series, with 11% morbidity and no mortality associated with the L-HIPEC [14]. That reinforces the notion that the morbidity and mortality associated with CRS and HIPEC is highly influenced by surgery extent.

This is a very small case series, and interpreting its results is somewhat limited. However, this multimodality treatment has been performed extensively in a Japanese institution and its results have shown a very acceptable toxicity and promising response rates, with long-term survival in selected patients [1]. We report here our group preliminary experience, with favorable results in subjects with very advanced gastric cancer and diffuse peritoneal metastases. A higher number of cases should confirm the validity of these results and provide more meaningful analyses.

## Conclusions

In conclusion, the association of L-HIPEC and BISIC has led to a good response in peritoneal disease extent in our initial experience and allowed radical procedures to be performed in individuals who were otherwise candidates for palliative chemotherapy.
